# Incidence rates of digestive cancers among U.S. military servicemen: Comparison with the rates in the general U.S. population

**DOI:** 10.1371/journal.pone.0257087

**Published:** 2021-09-03

**Authors:** Julie A. Bytnar, Craig D. Shriver, Kangmin Zhu

**Affiliations:** 1 John P. Murtha Cancer Center Research Program, Uniformed Services University of the Health Sciences and Walter Reed National Military Medical Center, Bethesda, Maryland, United States of America; 2 Henry M. Jackson Foundation for the Advancement of Military Medicine, Inc., Bethesda, Maryland, United States of America; 3 Department of Surgery, Uniformed Services University of the Health Sciences, Bethesda, Maryland, United States of America; 4 Department of Preventive Medicine and Biostatistics, Uniformed Services University of the Health Sciences, Bethesda, Maryland, United States of America; University of Cincinnati College of Medicine, UNITED STATES

## Abstract

**Background:**

Digestive cancers greatly contribute to the cancer burden in the United States. These cancers are more common among men and some are increasing among adults under age 50. Military population, which is dominantly male and young, and general populations differ in exposure to risk factors for these cancers. However, no studies have systematically investigated whether the incidence rates of these cancers differ between the two populations. This study aimed to compare incidence rates and trends of select digestive cancers between active-duty military and general populations in men aged 20–59 years.

**Methods:**

Data were from the Department of Defenses’ Automated Central Tumor Registry (ACTUR) and the National Cancer Institute’s Surveillance, Epidemiology, and End Results 9 (SEER-9) registries. Age-adjusted incidence rates of colorectal, stomach, liver, and pancreatic cancers among men aged 20–59 years during 1990–2013 were compared between the two populations. Stratified analyses by age were done for colorectal and stomach cancers. The joinpoint regression analysis was conducted to examine temporal trends for colorectal cancer.

**Results:**

The age-adjusted incidence rates of colorectal, stomach, liver, and pancreatic cancers were overall lower among active-duty than SEER (IRR = 0.86, 95% CI = 0.81–0.92; IRR = 0.65, 95% CI = 0.55–0.76; IRR = 0.39, 95% CI = 0.30–0.49; IRR = 0.51, 95% CI = 0.41–0.62, respectively). This was observed in the groups of both ages 20–39 and 40–59 years for stomach cancer, and in the group of ages 40–59 years for colorectal cancer. The incidence rates of colorectal cancer tended to decrease since 2008 in ACTUR.

**Conclusion:**

The incidence rates for selected digestive cancers overall were lower in the active-duty military population than the U.S. general population. This study highlights the need for more research enhancing our understanding of variations in these cancers between the two populations.

## Introduction

Digestive cancers include cancers of the intestinal tract (esophagus, stomach, small and large intestines, rectum, and anus) and the accessory digestive organs (liver, gallbladder, and pancreas). Colorectal cancer is the third most common cancer in incidence and mortality [1]. Liver and pancreatic cancers have both increased in incidence and mortality in recent decades and have low relative survival [1]. Together, digestive cancers account for 20% of cancers diagnosed and almost 30% of deaths among men each year [1].

Incidences rates of digestive cancers vary by age, gender, and time. Digestive cancers increase with age [2–5]. Most digestive cancers are more common among men with the incidence rates 2 to 3 times higher than those among women [1]. Temporal trends vary by cancer site and age. While the incidence of liver cancer has tripled [2] in the U.S. since the 1970s and pancreatic cancer [4] has similarly increased since 1994, the overall incidence rates of colorectal [3], and stomach [1] cancers have decreased over time. However, temporal trends of these cancers vary by age. For instance, while the incidence rates of colorectal cancer in the U.S. have been decreasing among people ages 50 or older, rates have been increasing among younger people in the past more than 20 years [6]. Increased incidence over time in younger people was also observed for stomach and pancreatic cancers [7].

Incidence of cancer is related to exposure to risk factors as well as detection of cancer such as that by cancer screening. In regard to the exposure to risk factors, stomach, liver, pancreatic, and colorectal cancers may be associated with tobacco use [8], alcohol use [9], diet [10–12], body composition [13], and physical activity [14] with relative impacts varying by cancer site. In addition, digestive cancers may be associated with some environmental and occupational risk factors, including radiation [15] and pesticides [16]. Regarding cancer detection, the incidence and mortality rates of colorectal cancer have been decreased in people ages 50 and older due to increased detection and subsequent treatment of precancerous lesions resulting from recommended colorectal cancer screening [17].

Active-duty military and general civilian populations have differences in health status and exposure to risk factors. The military population is healthier due to medical screenings and physical fitness assessments upon entrance to the military and during military service. On the other hand, enlisted service men have had higher prevalence of tobacco and alcohol use than men in the U.S. general population [18]. The military may be more likely to be exposed [19–24] to certain occupational risk factors such as radiation, air pollutants, solvents, and pesticides which have been linked to some digestive cancers compared to the general population.

Despite differences in factors related to cancer occurrence between the military and general populations, there has been very little research on the incidence of digestive cancers in the U.S. military. Two previous studies have shown that a lower incidence of colorectal cancer among men in the U.S. Air Force [25] and in the U.S. military overall [26] when compared to the U.S. general population. Neither evaluated other digestive cancers.

This study aimed to systematically investigate incidence rates of common digestive cancers. Specifically, we compared incidence rates of colorectal, stomach, liver, and pancreatic cancers among active-duty men to those in the U.S. general population, using the data from the Department of Defense’s (DoD) Automated Central Tumor Registry (ACTUR) and the National Cancer Institute’s Surveillance Epidemiology and End Results (SEER) program.

## Materials and methods

### Ethics statement

Use of the ACTUR data was approved by the institutional review board of Walter Reed National Military Medical Center. SEER data is publicly available. Patient consent was not required because data were not identifiable, and cancer is a reported disease in the United States.

### Data sources and study populations

ACTUR, which was established in 1986 to collect and track all cancer patients among DoD beneficiaries diagnosed or treated at military treatment facilities, was the data source for the U.S. military. Male active-duty patients ages 20 to 59 years were included in the study if they were diagnosed pathologically confirmed invasive colorectal, stomach, liver, and pancreatic cancers from 1990 through 2013. These cancer sites were chosen from all digestive cancers because they are the most common digestive cancers or have a high mortality rate [1], and had a relatively large number of cases in ACTUR. To prevent the potential of any impact due to incomplete reporting the initial years of ACTUR, Data before 1990 was not utilized. Through previously described consolidation procedures [26], only one summary record for each primary digestive cancer was included. The Defense Manpower Data Center (DMDC), which maintains demographic and military data for the DoD, as the data source for the annual number of active-duty service members.

Data from the SEER 9 registries was the source for the U.S. general population. The SEER 9 included nine areas (Atlanta, Connecticut, Detroit, Hawaii, Iowa, New Mexico, San Francisco-Oakland, Seattle-Puget Sound, and Utah) together representing approximately 10% of the U.S. population [27]. Men aged 20 to 59 years, who were diagnosed with colorectal, stomach, liver, or pancreatic cancer from 1990 through 2013 were included in the study. The annual U.S. population size from the U.S. Census was obtained through SEER [28]. The SEER data were consolidated according to the SEER*DMS User Manual and only one record was used for each primary cancer [29].

### Study variables

The following International Classification of Diseases for Oncology (ICD-O-3) topographic codes were used to define each cancer site: colorectal cancer C180, C182-209, stomach cancer C160-169, liver cancer C220-221, and pancreatic cancer C250-259 [30]. SEER guidelines were used to convert the earlier ICD-O codes used before 2001 in both data sources to the current ICD-O-3 system. Demographic characteristic included in this study were age at diagnosis (20–39 and 40–59) and race (White, Black, and other). The military service branches included the Army, Air Force, Navy, and Marine Corps.

### Statistical analysis

Age-adjusted incidence rates (per 100,000 person-years) and 95% confidence intervals (CIs) using the Tiwari method were calculated for each population [31]. The standard population used for age adjustment was the combined military population from 1990 to 2013, which gave more weight to the younger age groups with a large number of active-duty members for more stable rates.

Incidence rate ratio (IRR) and 95% CI were calculated for each cancer site to compare incidence rates between ACTUR and SEER. The comparisons were further stratified by age at diagnosis for colorectal cancer and stomach cancer, and by race and year of diagnosis for colorectal cancer. We did not do stratification by age for liver and pancreatic cancers and by race and year of diagnosis for stomach, liver, and pancreatic cancers because of small numbers of patients. For colorectal cancer, which had a larger number of patients, we then examined temporal trends by calculating the average annual percent change (AAPC) in incidence within each population using log-linear joinpoint regression [32]. All analyses were conducted using SAS (version 9.4) with a significance level set at *P* < .05 and the joinpoint regression analysis was conducted using the Joinpoint Regression program (version 4.9.0.0, NCI).

## Results

There were 1,102, 178, 78, and 97 cases with colorectal, stomach, liver and pancreatic cancers diagnosed, respectively, over 27,644,018 person-years in the ACTUR data. The corresponding numbers were 40,135, 7,524, 7,439, and 8,045 over 180,141,863 person-years in the SEER data. Table 1 shows the incidence rates in ACTUR and SEER. The incidence rates were significantly lower in ACTUR than SEER for colorectal (IRR = 0.86, 95% CI = 0.81–0.92), stomach (IRR = 0.65, 95% CI = 0.55–0.76), liver (IRR = 0.39, 95% CI = 0.30–0.49), and pancreatic (IRR = 0.0.51, 95% CI = 0.41–0.62) cancers, respectively.

**Table 1:** Incidence rates of selected digestive cancers in the U.S. active-duty military and U.S. general population, ages 20–59, 1990–2013.

	ACTUR^a^		SEER^b^		
	Count	Rate^c^ (95% CI)^d^	Count	Rate^c^ (95% CI)^d^	IRR^e^ (95% CI)^d^
**Colorectal**	1,102	3.99 (3.75–4.23)	40,135	4.63 (4.54–4.73)	0.86 (0.81–0.92)
**Stomach**	178	0.64 (0.55–0.75)	7,524	0.99 (0.95–1.04)	0.65 (0.55–0.76)
**Liver**	78	0.28 (0.22–0.35)	7,439	0.73 (0.69–0.76)	0.39 (0.30–0.49)
**Pancreas**	97	0.35 (0.28–0.43)	8,045	0.69 (0.66–0.72)	0.51 (0.41–0.62)

^a^ ACTUR, Automated Central Tumor Registry.

^b^ SEER, Surveillance, Epidemiology and End Results.

^c^ Age-adjusted rate per 100,000 person-years (the active-duty military population, 1990–2013, as the standard population).

^**d**^ 95% confidence interval.

^e^ Incidence Rate Ratio comparing the rate in ACTUR to that in SEER.

When data were analyzed by age for colorectal and stomach cancers (Table 2), the incidence of cancer among individuals aged 20–39 years was significantly lower in ACTUR than SEER for colorectal (IRR = 0.77, 95% CI = 0.70–0.85) and stomach (IRR = 0.62, 95% CI = 0.50–0.77) cancers, respectively. In the group of ages 40–59, while the rate of stomach cancer was lower in ACTUR than SEER (IRR = 0.68, 95% CI = 0.53–0.84), the difference between the two populations was not significant for colorectal cancer (IRR = 0.95, 95% CI = 0.87–1.04). Table 3 shows the results stratified by race. Incidence rates were significantly lower in ACTUR than SEER among White men for colorectal cancer (IRR = 0.83, 95% CI = 0.77–0.89).

**Table 2:** Incidence rates of colorectal and stomach cancers by age in the U.S. active-duty military and U.S. general population, ages 20–59, 1990–2013.

	ACTUR^a^			SEER^b^			
	Count	Person-Years	Rate^c^ (95% CI)^d^	Count	Person-Years	Rate^c^ (95% CI)^d^	IRR^e^ (95% CI)^d^
**Colorectal**							
20–39 years	514	24,684,988	2.08 (1.91–2.27)	3,538	96,438,912	2.69 (2.60–2.79)	0.77 (0.70–0.85)
40–59 years	588	2,959,030	19.87 (18.30–21.54)	36,597	83,702,951	20.84 (20.47–21.22)	0.95 (0.87–1.04)
**Stomach**							
20–39 years	95	24,684,988	0.38 (0.31–0.47)	801	96,438,912	0.62 (0.57–0.66)	0.62 (0.50–0.77)
40–59 years	83	2,959,030	2.80 (2.23–3.48)	6,723	83,702,951	4.14 (3.97–4.31)	0.68 (0.53–0.84)

^a^ ACTUR, Automated Central Tumor Registry.

^b^ SEER, Surveillance, Epidemiology and End Results.

^c^ Age-adjusted rate per 100,000 person-years (the active-duty military population, 1990–2013, as the standard population).

^**d**^ 95% confidence interval.

^e^ Incidence Rate Ratio comparing the rate in ACTUR to that in SEER.

**Table 3:** Incidence rates of colorectal cancer by race in the U.S. active-duty military and U.S. general population, ages 20–59, 1990–2013.

	ACTUR^a^			SEER^b^			
	Count	Person-Years	Rate^c^ (95% CI)^d^	Count	Person-Years	Rate^c^ (95% CI)^d^	IRR^e^ (95% CI)^d^
**Colorectal**							
White	770	20,723,750	3.65 (3.40–3.92)	30,319	139,110,100	4.41 (4.30–4.52)	0.83 (0.77–0.89)
Black	239	5,012,364	5.09 (4.45–5.81)	5,192	20,404,072	5.48 (5.21–5.77)	0.93 (0.80–1.07)
Other race	77	1,907,904	4.43 (3.49–5.54)	4,350	20,627,691	5.07 (4.79–5.37)	0.87 (0.68–1.10)

^a^ ACTUR, Automated Central Tumor Registry.

^b^ SEER, Surveillance, Epidemiology and End Results.

^c^ Age-adjusted rate per 100,000 person-years (the active-duty military population, 1990–2013, as the standard population).

^**d**^ 95% confidence interval.

^e^ Incidence Rate Ratio comparing the rate in ACTUR to that in SEER.

**Fig 1:**
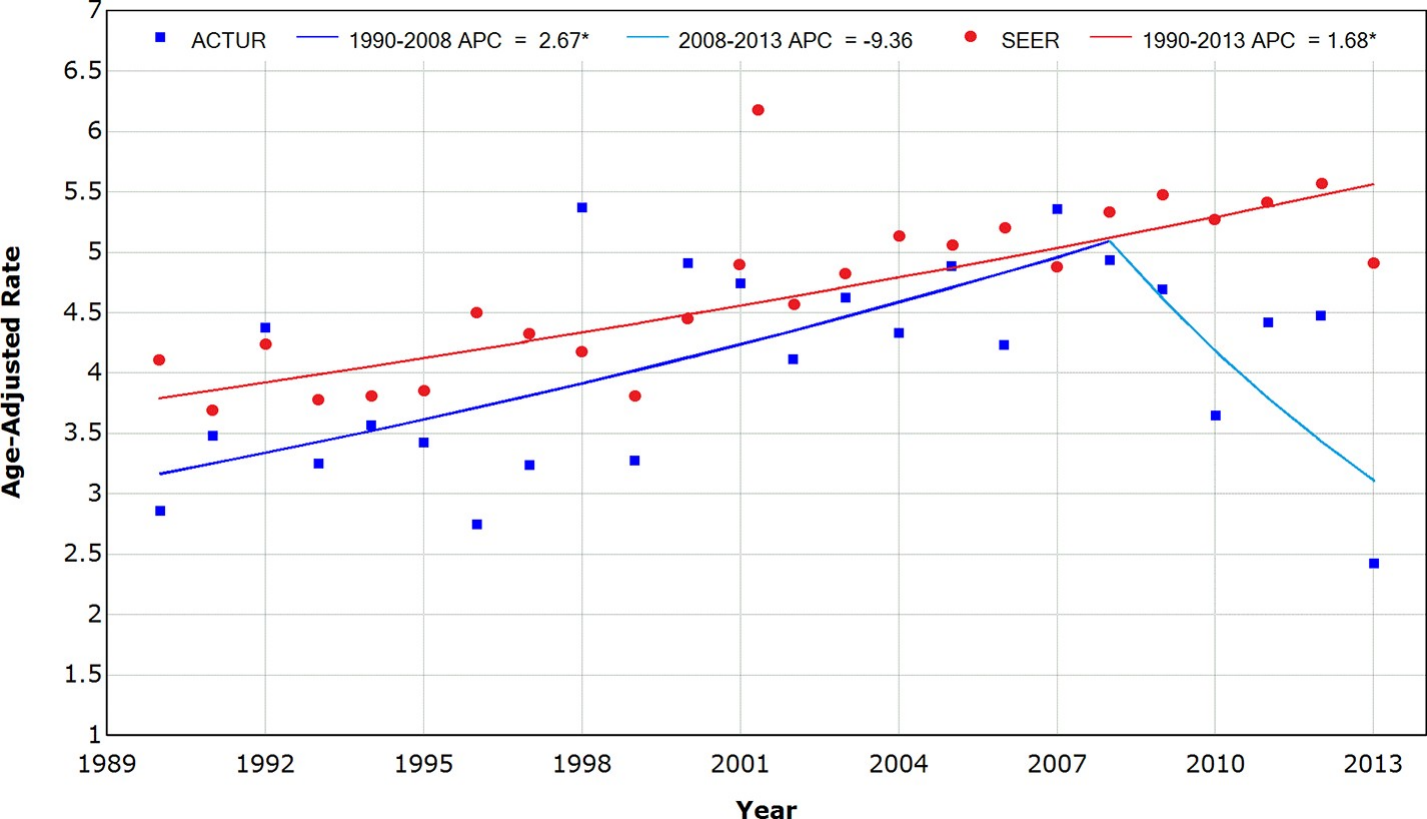
Trends in colorectal cancer incidence rates in the U.S. military population (ACTUR) and the U.S. general population (SEER), 1990–2013. * Indicates that the Annual Percent Change (APC) is significantly different from zero at the alpha = 0.05 level.

Fig 1 shows the trends in colorectal cancer incidence over the study period by study population. The best fitting model showed a significant increase of 2.67% in the average annual percentage change (AAPPC) over the entire study period for SEER whereas the corresponding AAPC was significant through 2008 (1.68%) for ACTUR. From 2008 through 2013, incidence appeared to decrease for ACTUR, but the AAPC (-9.36%) was not statistically significant.

## Discussion

Our study showed that the incidence rates of colorectal, stomach, liver, and pancreatic cancers among men were significantly lower in the U.S. active-duty military population than the general U.S. population, respectively. The lower incidence rates among the active-duty members were observed among men aged 20–39 years for the two cancer sites included in stratified analysis (colorectal and stomach). In the older group (45–59 years), it was observed for stomach cancer only. The analyses by race showed a significantly lower rate of colorectal cancer in the military population than the general population among White men. Temporal trends in the incidence of colorectal cancer differed between the active-duty military and general populations. While the incidence of colorectal cancer was increased in the general population over the entire study period, it only increased through 2008 and tended to decrease for 2008–2013 in the military population.

Healthier status of active-duty members might be related to the lower incidence rates of these cancers. Active-duty members must meet rigorous medical and physical standards for entering military service. During their service, they are engaged in more physical activities, must pass routine physical fitness assessments, and must maintain healthy body weights so that they are less likely to be overweight or obese than the general U.S. population. It is known that increased physical activity levels [14] and healthy body weights [13] are protective factors for colorectal, stomach, liver, and pancreatic cancers. In addition, service members receive free care and thus may be more likely to have a cancer precursor detected and treated, reducing the possibility of cancer occurrence. The other possible factor for the lower incidence is under-reporting of cases to ACTUR from some small military treatment facilities, which might have fewer resources than larger facilities for reporting. Nevertheless, other studies in the active-duty ACTUR population found higher incidence rates of prostate, breast, thyroid, and melanoma malignancies in the ACTUR than in the U.S. general population [26,33,34], which suggests that the effects of underreporting might be limited.

In the stratified analyses on colorectal and stomach cancers, no significant difference between the military and U.S. general populations was observed for colorectal cancer among men aged 40–59 years. The reasons for this are not clear but may result from synthetic effects of multiple factors. While healthier status, more physical activity, and lower obesity rate may be protective, the military population may also be exposed to factors that increase the risk of these cancers. For example, U.S. military members may take in fewer fruits and vegetables than their civilian counterparts [35], which is a risk factor for colorectal cancer [36]. They may also have more exposures to environmental and occupational risk factors, such as radiation [37], metals and chemical materials [38], which may increase the risk of developing digestive cancers. Although we do not have access to the information regarding length of service, individuals in the older age group (40–59 years) may have had a longer length of military service, and therefore more cumulative military-related exposures to the risk factors, which may counteract the effects of healthier status and protective factors. In addition, longer military service allows for a longer follow-up time to identify cancer occurrence in the ACTUR database. Thus, the lower incidence in ACTUR than the general population was no longer observed for the older group.

Although a lower incidence rate of colorectal cancer in the military population than the general population was significant only among White men, the similar tendency was observed for Black and other races. The insignificance in these groups might be related to smaller numbers of patients, which led to a lower study power to identify the difference between the two populations.

It is not clear why the incidence of colorectal cancer increased over the entire study period in the general population, but only through 2008 in the military. After 2008, the incidence insignificantly decreased in the military. While more data are needed to demonstrate whether this decreasing trend continues and is significant in the military, there is a possibility that active-duty members who receive free medical care might become more aware of colorectal examination over time, were more likely to have colorectal lesions detected and removed, and thus the incidence of colorectal cancer was further lower in more recent years.

This study has some limitations. First, due to relatively small numbers of ACTUR cases, the analyses stratified by age were not feasible for liver and pancreatic cancers and the analysis stratified by race and trend analysis could be conducted only for colorectal cancer. Second, ethnicity and population movement (such as population changes over time due to recruitment to and retirement from the military) may have affected the results if they were related to cancer occurrence. Due to lack of complete data, we were unable to assess their effects. Third, we cannot exclude the possibility of under-reporting in ACTUR because some small military treatment facilities might have fewer resources to report their cases. However, the potential effects of this possibility might be limited since our previous studies on prostate, breast, thyroid, and melanoma cancers found higher incidence rates in the ACTUR than in the U.S. general population [26,33,34]. Further research with more cases in the future are warranted.

In conclusion, we found that active-duty military servicemen had lower overall incidence rates of colorectal, stomach, liver, and pancreatic cancers than the U.S. general population. These findings provide clues for further research on factors associated with these cancers.
